# Chromosomal Instability in *BRAF* Mutant, Microsatellite Stable Colorectal Cancers

**DOI:** 10.1371/journal.pone.0047483

**Published:** 2012-10-22

**Authors:** Catherine E. Bond, Aarti Umapathy, Ron L. Buttenshaw, Leesa Wockner, Barbara A. Leggett, Vicki L. J. Whitehall

**Affiliations:** 1 Conjoint Gastroenterology Laboratory, The Queensland Institute of Medical Research, Brisbane, Australia; 2 The School of Medicine, The University of Queensland, Brisbane, Australia; 3 Pathology Queensland, Clinical and Statewide Services, Queensland Health, Brisbane, Australia; 4 Statistics Unit, The Queensland Institute of Medical Research, Brisbane, Australia; 5 The Royal Brisbane and Women’s Hospital, Queensland, Australia; University of North Carolina School of Medicine, United States of America

## Abstract

The *BRAF* oncogene is mutated in 15% of sporadic colorectal cancers. Approximately half of these *BRAF* mutant cancers demonstrate frequent frameshift mutations termed microsatellite instability (MSI), but are diploid and chromosomally stable. *BRAF* wild type cancers are typically microsatellite stable (MSS) and instead acquire chromosomal instability (CIN). In these cancers, CIN is associated with a poor outcome. *BRAF* mutant cancers that are MSS, typically present at an advanced stage and have a particularly poor prognosis. We have previously demonstrated clinical and molecular similarities between MSS cancers with or without a *BRAF* mutation, and therefore hypothesised that CIN may also be frequent in *BRAF* mutant/MSS cancers. *BRAF* mutant/MSS (n = 60), and *BRAF* wild type/MSS CRCs (n = 90) were investigated for CIN using loss of heterozygosity analysis over twelve loci encompassing chromosomal regions 5q, 8p, 17p and 18q. CIN was frequent in *BRAF* mutant/MSS cancers (41/57, 72%), which was comparable to the rate found in *BRAF* wild type/MSS cancers (74/90, 82%). The greatest loss in *BRAF* mutant/MSS cancers occurred at 8p (26/44, 59%), and the least at 5q (19/49, 39%). CIN in *BRAF* mutant/MSS cancers correlated with advanced stage (AJCC III/IV: 15/17, 88%; p = 0.02); showed high rates of co-occurrence with the CpG Island Methylator Phenotype (17/23, 74%); and CIN at 18q and 8p associated with worse survival (p = 0.02, p<0.05). This study demonstrates that CIN commonly occurs in advanced *BRAF* mutant/MSS colorectal cancers where it may contribute to poorer survival, and further highlights molecular similarities occurring between these and *BRAF* wild type cancers.

## Introduction

Sporadic colorectal cancer (CRC) is a diverse disease which results from the progression of differing types of precursor lesions that are molecularly and morphologically distinct. These lesions acquire genetic alterations associating with one of at least two recognized molecular pathways leading to tumorigenesis. The ‘traditional pathway’ is the most well characterized and involves the progression of a conventional type adenoma that may acquire mutation or loss of *APC*, mutation of *KRAS* and *p53*, and chromosomal instability prior to the formation of a carcinoma [1] which is typically *BRAF* wild type. The more recently described ‘serrated’ pathway involves the progression of a serrated lesion to cancer [2], [3], [4], [5]. This is accompanied by an early mutation of the *BRAF* oncogene [6], [7], and acquisition of the CpG Island Methylator Phenotype (CIMP) which involves widespread promoter hypermethylation and subsequent silencing of key tumour suppressor genes [8], [9].

*BRAF* is an integral component of the mitogen-activated protein kinase (MAPK) signalling cascade which promotes cellular proliferation and anti-apoptotic effects [10]. The *BRAF* mutation is considered a marker for the serrated pathway and is found in approximately 10–15% of CRC [11], including the majority of those showing CIMP. Approximately half of these cancers will have hypermethylation and silencing of a DNA mismatch repair gene, *MLH1*, due to CIMP [8], [12]. This results in multiple mutations within DNA repeat tracts, termed microsatellite instability (MSI) [13]. These MSI, *BRAF* mutated cancers have previously been well described as diploid [14], [15], [16], more commonly occurring in older females and the proximal colon, are often mucin producing and poorly differentiated [2], [15], [17], [18]. The remaining half of the *BRAF* mutant lesions of the serrated pathway that do not have methylation of *MLH1*, are microsatellite stable (MSS) [19], [20]. In contrast to MSI cancers, these have not been well characterized.

Interestingly, the two *BRAF* mutant subgroups that differ by microsatellite instability status, confer significantly contrasting prognoses. Whilst *BRAF* mutant/MSI cancers correlate with an excellent patient outcome, *BRAF* mutant/MSS cancers are associated with a very poor outcome that is even worse than the *BRAF* wild type/MSS cancers arising via the ‘traditional pathway’ [20], [21], [22]. The molecular mechanisms underlying this disparity are unknown.

Two distinct forms of genomic instability are known to occur in CRC: MSI and chromosomal instability (CIN) [23], [24]. MSI affects genomic integrity at the DNA level and develops at the polyp/carcinoma transition of *BRAF* mutant serrated polyps which silence *MLH1* by DNA methylation through the CIMP phenotype [13], [25]. Alternatively, CIN which affects approximately 70% of CRCs [18], acts on a wider genomic scale as it refers to losses and/or gains of whole or part chromosomal regions, and has been associated with poorer survival [16], [26], [27], [28]. CIN develops in conventional adenomas which are *BRAF* wild type and progress towards malignancy via the ‘traditional pathway’. Loss of heterozygosity (LOH), which describes either loss or reduction of one of the two parental alleles at a particular chromosomal location, indicates the presence of CIN. The chromosomal regions 18q, 17p, 5q, and 8p, which harbour key tumour suppressor genes, have been found to show extensive LOH in CRC [1], [23].

It is unclear whether *BRAF* mutant serrated polyps which become malignant but do not methylate *MLH1* and do not develop MSI, manifest chromosomal instability. For example, some recent studies have suggested that CIMP and CIN are mutually exclusive [29], [30]. Whilst this is concordant with the diploid nature of *BRAF* mutant/MSI serrated pathway cancers with CIMP which progress via a high mutational rate, it does not explain how *BRAF* mutant/MSS serrated pathway cancers progress and especially the fact that their prognosis is particularly poor [16], [29], [30], [31], [32].

We hypothesized that although CIN may be mutually exclusive with MSI, it is not mutually exclusive with CIMP and may be common in *BRAF* mutant/MSS cancers. MSI cancers have been excluded for LOH analysis in this and previous studies due to the documented low levels of CIN previously found, and because the degree of instability at microsatellite markers present in MSI cancers does not allow for informative assessment [26], [30], [31], [32].

## Materials and Methods

### Ethics Statement

Written, informed consent was obtained from each patient involved in this study. This research was approved by the Royal Brisbane and Women’s Hospital and Bancroft Human Research Ethics Committees.

### Patient Samples

1052 sporadic CRC and matched normal mucosa samples were obtained after surgical excision at the Royal Brisbane and Women’s Hospital (RBWH), Queensland, Australia. Clinical data, including patient age of onset and gender, anatomical location of cancer, cancer stage and patient outcome were collected where possible from review of patient charts, pathology reports or the Queensland Death Registry. Anatomical location was specified as either proximal (proximal to splenic flexure) or distal. Cancer stage was classified according to both the American Joint Committee on Cancer (AJCC) and Tumour Node Metastases (TNM) staging systems [33].

#### MSI status, CIMP, BRAF, KRAS and p53 mutation detection

Samples had previously been investigated for MSI according to the National Cancer Institute’s 5 marker panel consisting of two mononucleotide markers, BAT25, BAT26; and three dinucleotide markers, D5S346, D2S123 and D17S250 [34]. MSI was classified if at least one of the mononucleotide markers and at least one other marker (either mononucleotide or dinucleotide) was positive [34], [35]. CIMP was previously investigated using MethyLight with a five marker panel consisting of CACNA1G, IGF2, NEUROG1, RUNX3, SOCS1 [36], [37]_ENREF_35_ENREF_35. Cancers were considered CIMP positive if 3 or more markers were methylated. All samples had been screened for the *BRAF* V600E (a1796t) mutation using an allelic discrimination assay, and *KRAS* mutations in codons 12 and 13 using Sequenom MassARRAY technology [37]. The incidence of *p53* mutation across exons 4 to 8 has previously been reported for all cancers included in this study [37].

### Loss of Heterozygosity Analysis

Chromosomal instability (CIN) was assessed by loss of heterozygosity (LOH) analysis over a total of 12 loci covering 4 chromosomal regions known to harbour key tumour suppressor genes implicated in CRC. These were centred over 5q22.2 (containing the *APC* gene); 8p22 (linked to a CRC suppressor region); 17p13.1 (spanning the *p53* locus), and 18q (for *SMAD2*, *SMAD4* and *DCC* loci). Three markers were investigated over each of the four regions (5q: D5S346, D5S1466, D5S489; 8p: D8S258, D8S254, D8S1121; 17p: 17S261, 17S578, D17S926; and 18q: D18S55, D18S460, D18S487). Primer sequences for each of the markers were obtained from the Ensembl website (http://www.ensembl.org/index.html). (For details of LOH PCR reactions refer to Table 1 in Supplementary Data S1). Paired normal and cancer PCR products were electrophoresed on a 5% polyacrylamide gel and observed radiologically.

**Table 1:** Clinicopathological and Molecular Characteristics of the *BRAF*mut/MSS and *BRAF*wt/MSS cohorts.

Characteristic:	*BRAF* mut/MSS (Serrated)	*BRAF* wt/MSS (Traditional)	P value	
**N**	60	90	–	
**Stage AJCC I**	2/31 (6.5%)	11/70 (15.7%)	0.33	
**Stage AJCC II**	12/31 (38.7%)	26/70 (37.1%)	1.0	
**Stage AJCC III**	11/31 (35.5%)	20/70 (28.6%)	0.65	
**Stage AJCC IV**	6/31 (19.4%)	13/70 (18.6%)	1.0	
**Proximal Location**	33/48 (68.8%)	25/82 (30.5%)	**<0.0001**	
**Av Age of Onset (yrs)**	67.3	68.2	0.68	
**Female Gender**	31/60 (51.7%)	39/90 (43.3%)	0.32	
***p53*** ** mutation**	21/57 (36.8%)	42/90 (46.7%)	0.31	
**CIMP high**	27/47 (57.4%)	3/79.(3.8%)	**<0.0001**	
***KRAS*** ** mutation**	0	41/90 (45.6%)	–	

LOH at individual loci was scored if one of the two alleles of the cancer sample had at least a 40% reduction in intensity compared to its paired normal sample. A polymorphic marker where the two parental alleles were able to be visualised was considered informative. The overall extent of CIN was observed over the 12 loci, and CIN positivity (CIN) was assigned if at least 1 informative marker was observed with LOH. CIN negativity was scored if at least 40% (5 of 12) of markers were informative, and none of these demonstrated LOH. For analysis of CIN at individual regions, the 3 loci were observed and scored as CIN if at least one showed LOH, and CIN negative if 2 of the 3 loci were informative and neither demonstrated LOH.

The fractional allelic loss (FAL) has previously been described [38]. This approach was utilised as further analysis of the presence of CIN, where the total number of allelic loss events was divided by the total number of informative markers per sample and per cohort. We used this to verify the overall rate of allelic loss, and correlations with FAL and stage at presentation, cancer location and gender, and molecular features of *p53* and *KRAS* mutation, and CIMP were determined.

### Statistical Analysis

Significant relationships between categorical data were assessed using Fisher’s Exact Test (SPSS version 19; Graphpad software). Differences in continuous data were assessed using t-tests. Cox proportional hazard models were utilised to investigate the effects of various factors on survival rate where death due to cancer was taken to be the event of interest. A log rank test was used to assess the equivalence of distributions when applicable. P-values ≤0.05 were considered significant.

## Results

### BRAF Mutation in MSS Colorectal Cancers

Of 1052 cancers, 128 (12.2%) had a *BRAF* mutation. Out of these *BRAF* mutants, 60 (4.7%) were MSS (*BRAF*mut/MSS) and formed the experimental cohort. This was compared to a randomly selected control cohort of 90 MSS, *BRAF* wild type cancers (*BRAF*wt/MSS). *BRAF*mut/MSS cancers were predominantly located in the proximal colon (33/48, 69%) compared to the *BRAF*wt/MSS cancers (25/82, 30.5%) (p<0.0001). These proximal, *BRAF* mut/MSS cancers more commonly arose in females (22/33, 66.7% females; p = 0.01) and there was a non-significant trend toward older age (68.6 years for proximal cancers versus 63.1 years for distal cancers; p = 0.1). The *BRAF*mut/MSS cancers showed no difference in rate of CIMP in the proximal (17/27, 63.0%) versus distal bowel (6/12, 50%), rate of *p53* mutation in the proximal (14/31, 45.2%) or distal bowel (6/14, 42.9%), or in stage at diagnosis in the proximal (10/21, 47.6%) versus distal bowel (4/9, 44.4%).

Overall, the BRAFmut/MSS cohort did not differ by age of onset, gender distribution or stage at diagnosis to the *BRAF*wt/MSS cohort (Table 1). Molecularly, the *BRAF*mut/MSS cancers did not differ from *BRAF*wt/MSS cancers with extent of *p53* mutation (21/57, 37% versus 42/90, 47%), but were significantly more likely to demonstrate CIMP (27/47, 57% versus 3/79, 4%) (p<0.0001) (Table 1). In the *BRAF*wt/MSS cohort, a *KRAS* mutation was present in 41/90 (46%) cancers (Table 1).

### Overall Frequency of Chromosomal Instability (CIN)

The *BRAF*mut/MSS and *BRAF*wt/MSS cancer cohorts displayed highly informative markers for LOH at 95% (57 of 60) and 100% (90 of 90) respectively.

Both cohorts demonstrated a high rate of CIN regardless of *BRAF* mutation status, with 41/57 (72%) *BRAF* mutant, and 74/90 (82%) *BRAF* wild type showing CIN (Table 2). The fractional allelic loss (FAL) [38] which describes the frequency of allelic loss events amongst the total number of informative events, was analysed as a further method of quantifying the overall degree of CIN within a cohort. *BRAF*mut/MSS cancers showed a slightly lower average FAL compared to *BRAF*wt/MSS cancers (132/428 = 0.308, versus 286/745 = 0.384 respectively), but this difference was not statistically significant (p = 0.1) (Table 3).

**Table 2:** Molecular Characteristics of Cohorts Relative to Presence or Not of Chromosomal Instability (CIN).

Molecular Characteristic:	*BRAF* mut/MSS (Serrated)	P value	*BRAF* wt/MSS (Traditional)	P value	P value between cohorts
**N**	60		90		
**Overall CIN+**	41/57 (71.9%)		74/90 (82.2%)		0.16
**5q CIN+**	19/49 (38.8%)		43/80 (53.8%)		0.11
**8p CIN+**	26/44 (59.1%)		39/72 (54.2%)		0.70
**17p CIN+**	23/43 (53.5%)		43/65 (66.2%)		0.23
**18q CIN+**	19/48 (39.6%)		50/83 (60.2%)		**0.03**
***p53*** ** mut | CIN+** *	16/37 (43.2%)	0.23	38/70 (54.2%)	**0.01**	0.31
***p53*** ** mut | CIN−** *	4/17 (23.5%)		4/20 (20.0%)		1.0
***p53*** ** mut|5q CIN+**	7/19 (36.8%)	1.0	24/43 (55.8%)	**0.04**	0.27
***p53*** ** mut|5q CIN−**	9/27 (33.3%)		12/37 (32.4%)		1.0
***p53*** ** mut|8p CIN+**	10/24 (41.7%)	0.75	23/39 (59.0%)	**0.02**	0.21
***p53*** ** mut|8p CIN−**	6/17 (35.3%)		10/33 (30.3%)		0.76
***p53*** ** mut|17p CIN+**	14/22 (63.6%)	**0.03**	26/43 (60.5%)	**0.02**	1.0
***p53*** ** mut|17p CIN−**	5/18 (27.8%)		6/22 (27.3%)		1.0
***p53*** ** mut|18q CIN+**	8/17 (47.1%)	0.54	28/50 (56.0%)	**0.01**	0.58
***p53*** ** mut|18q CIN−**	10/28(35.7%)		9/33 (27.3%)		0.58
***KRAS*** ** mut|CIN+**	–		32/74 (43.2%)	0.41	–
***KRAS*** ** mut|CIN−**	–		9/16 (56.3%)		–
**CIN+ | CIMP high**	18/25 (72.0%)	0.75	2/3 (66.7%)	0.39	1.0
**CIN+ | CIMP nil/low**	13/20 (65.0%)		65/76 (85.5%)		**0.05**

CIN+ was indicated by the presence of LOH. This was scored for overall CIN+ if a cancer had 1 of the 12 markers showing greater than 40% loss in intensity of one allele compared to its paired normal. CIN- was assigned if 40% (5 of 12) markers were informative and none of these showed LOH. For individual regions, CIN+ was scored if 1 marker showed LOH, and CIN- if 2of the 3 markers were informative and neither had LOH. CIMP high was scored if 3 of the 5 markers were positive for methylation.

* CIN and corresponding *p53* mutational status was analysed using CIN data at the 5q, 8p and 18q loci only.

### CIN at Individual Chromosomal Regions

The *BRAF*mut/MSS cancers showed CIN at the highest frequency at the 8p chromosomal region (26 of 44, 59%), and the least at the 5q region (19 of 49, 39%). The *BRAF*wt/MSS cancers had the highest frequency of CIN at 17p (43 of 65, 66%), and the lowest rates of CIN+ at 5q (43 of 80, 54%) and 8p (39 of 72, 54%) (Table 2).

The *BRAF*wt/MSS cancers had a higher frequency of CIN events compared to the *BRAF*mut/MSS cancers occurring at the individual chromosomal regions of 5q, 17p and 18q. This relationship was statistically significant at 18q, with 60% (50 of 83) of *BRAF*wt/MSS cancers showing CIN, compared to 40% (19 of 48) of *BRAF*mut/MSS cancers with CIN at this loci (p = 0.03) (Table 2).

### p53 Mutation Rate and Presence of CIN

Mutation of *p53* was correlated with deletion of the *p53* locus (chromosome 17p) (Table 2). The frequency of *p53* mutation in relation to CIN was therefore investigated with the exclusion of 17p data in order to avoid over-estimating an association that may be driven by inclusion of LOH positive markers surrounding the *p53* locus. Based on chromosome 5q, 8p and 18q loci; in the *BRAF*wt/MSS cohort, 38/70 (54%) of CIN cancers had a concurrent *p53* mutation, compared to only 4/20 (20%) of CIN-negative cancers (p = 0.01). The *BRAF* mutant/MSS cancers demonstrated a similar but non-significant trend, with 16/37 (43%) CIN cancers having a *p53* mutation compared to only 4/17 (24%) CIN-negative cancers (Table 2). Within the *BRAF*wt/MSS cohort, LOH at all individual chromosomal regions significantly correlated with a higher incidence of *p53* mutation (Table 2). Average FAL per cohort demonstrated a higher rate of loss coinciding with a *p53* mutation compared to those wild type for *p53*, significantly so for *BRAF*wt/MSS cancers (p = 0<0.001) (Table 3).

### KRAS Mutation Rate and Presence of CIN in BRAFwt/MSS Cancers

*BRAF* and *KRAS* mutations were confirmed to be mutually exclusive. The *BRAF*wt/MSS cancers had a 46% (41 of 90) *KRAS* mutation rate (Table 1). There was no significant association between a *KRAS* mutation and CIN in these cancers, with a *KRAS* mutation present in 43% (32 of 74) CIN cancers, and in 56% (9 of 16) CIN-negative cancers (Table 2). Interestingly of the CIN cancers, the average FAL was significantly lower in *KRAS* mutant compared to *KRAS* wild type cancers (p = 0.045) (Table 3).

### CpG Island Methylator Phenotype (CIMP) and CIN in BRAFmut/MSS Cancers

CIMP was present in 57% (27 of 47) *BRAF*mut/MSS cancers, whilst only 4% (3 of 79) *BRAF*wt/MSS cancers displayed CIMP (Table 1). Interestingly, a substantial proportion of *BRAF*mut/MSS cancers demonstrated both CIMP and CIN, where 72% of the CIMP high *BRAF*mut/MSS cancers showed CIN (Table 2).

### CIN and Clinicopathological Data

The presence of CIN did not affect age of onset and gender distributions (Table 2 in Supplementary data S1). The rate of CIN did not vary regardless of whether the cancer originated from the proximal or distal colon in either cohort, with frequency of allelic loss (FAL) in the *BRAF*mut/MSS cancers at 0.33 and 0.32; and in *BRAF*wt/MSS at 0.33 and 0.42 for proximal and distal cancers respectively (Table 3; Table 2 in Supplementary data S1).

In *BRAF*mut/MSS cancers, CIN significantly associated with advanced stage, occurring in 15 of 17 (88%) stage III/IV cancers compared to 6 of 13 (46%) stage I/II cancers (p = 0.02) (Table 4). Average FAL scores were consistent with this finding, with increasing FAL correlating with advancing stage in *BRAF*mut/MSS CIN cancers (p = 0.03) (Table 3). By contrast, the *BRAF*wt/MSS group had high levels of CIN at both late and early stages of presentation with 27/33 (82%) stage III/IV, and 31/37 (84%) stage I/II CIN cancers (Table 4). This was also demonstrated by similar FAL rates found in the *BRAF*wt/MSS late compared to early stage cancers (Table 3).

**Table 3:** Fractional Allelic Loss (FAL) describing the number of loss events over the total number of informative events for a sample per cohort.

Feature	N	BRAF mut/MSS Av Fractional Allelic Loss	P Value	N	BRAF wt/MSS Av Fractional Allelic Loss	P Value	P Value between cohorts
**Overall**	56	0.304	–	90	0.386	–	0.1
**AJCC Stage I+ II**	13	0.132	**0.01**	37	0.396	0.82	**0.005**
**AJCC St. III + IV**	16	0.362		33	0.412		0.55
**AJCC Stage I**	2	0.100	**0.03**	11	0.347	0.92	0.36
**AJCC Stage II**	11	0.138		26	0.417		**0.006**
**AJCC Stage III**	10	0.283		20	0.415		0.28
**AJCC Stage IV**	6	0.492		13	0.407		0.44
**Proximal Loc.**	30	0.333	0.91	25	0.325	0.19	0.92
**Distal Location**	15	0.323		57	0.416		0.25
**Female Gender**	28	0.225	**0.04**	39	0.392	0.86	**0.02**
**Male Gender**	28	0.383		51	0.381		0.98
***P53*** ** Mutant** *	20	0.375	0.17	42	0.491	**<0.001**	0.14
***P53*** ** Wild Type** *	36	0.260		48	0.274		0.83
**CIMP High**	24	0.240	0.35	3	0.444	0.81	0.26
**CIMP Low/Nil**	20	0.321		76	0.404		0.29
***Kras*** ** Mutant**	–	–	–	41	0.320	**0.045**	**–**
***Kras*** ** Wild Type**	–	–		49	0.441		

* Average FAL and corresponding *p53* mutational status was analysed using CIN data at the 5q, 8p and 18q loci only.

**Table 4:** CIN Positivity and Clinicopathological AJCC Staging of Cancer Cohorts.

	BRAF mut/MSS (Serrated)	P value	BRAF wt/MSS (Traditional)	P value	P value between cohorts
***Overall CIN+ | AJCC Stage:***					
Stage I	1/2 (50.0%)	**0.046**	8/11 (72.7%)	0.41	1.0
Stage II	5/11 (45.5%)		23/26 (88.5%)		**0.01**
Stage III	9/11 (81.8%)		15/20 (75%)		1.0
Stage IV	6/6 (100%)		12/13 (92.3%)		1.0
Stages I + II/CIN+	6/13 (46.2%)	**0.02**	31/37 (83.8%)	1.0	**0.02**
Stages III + IV/CIN	15/17 (88.2%)		27/33 (81.8%)		0.70
***5q CIN+ and Stage:***					
Stage I	0/1 (0%)	0.16	5/8 (62.5%)	0.95	0.44
Stage II	2/12 (16.7%)		14/25 (56.0%)		**0.04**
Stage III	3/11 (27.3%)		9/17 (52.9%)		0.25
Stage IV	3/4 (75.0%)		8/13 (61.5%)		1.0
Stages I + II	2/13 (15.4%)	0.22	19/33 (57.6%)	1.0	**0.02**
Stages III + IV	6/15 (40.0%)		17/30 (56.7%)		0.35
***8p CIN+ and Stage:***					
Stage I	1/1 (100%)	0.37	3/9 (33.3%)	0.47	0.40
Stage II	2/5 (40.0%)		12/18 (66.7%)		0.34
Stage III	4/9 (44.4%)		10/18 (55.6%)		0.70
Stage IV	5/6 (83.3%)		6/11 (54.5%)		0.33
Stages I + II	3/6 (50.0%)	1.0	15/27 (55.6%)	1.0	1.0
Stages III + IV	9/15 (60.0%)		16/29 (55.2%)		1.0
***17p CIN+ and Stage:***					
Stage I	0/2 (0%)	0.052	4/6 (66.7%)	0.94	0.43
Stage II	2/6 (33.3%)		16/23 (69.6%)		0.16
Stage III	4/8 (50.0%)		10/13 (76.9%)		0.35
Stage IV	5/5 (100%)		6/9 (66.7%)		0.26
Stages I + II	2/8 (25.0%)	0.08	20/29 (69.0%)	1.0	**0.04**
Stages III + IV	9/13 (69.2%)		16/22 (72.7%)		1.0
***18q CIN+ and Stage:***					
Stage I	1/2 (50.0%)	0.09	7/11 (63.6%)	0.87	1.0
Stage II	1/9 (11.1%)		15/22 (68.2%)		**0.006**
Stage III	6/9 (66.7%)		10/18 (55.6%)		0.69
Stage IV	3/5 (60.0%)		8/13 (61.5%)		1.0
Stages I + II	2/11 (18.2%)	**0.04**	22/33 (66.7%)	0.61	**0.01**
Stages III + IV	9/14 (64.3%)		18/31 (58.1%)		0.75
***Overall CIN+|TNM Stage:***					
T1	0	0.83	3/5 (60.0%)	0.59	–
T2	2/3 (66.7%)		6/7 (85.7%)		1.0
T3	13/20 (65.0%)		39/47 (82.0%)		0.12
T4	6/7 (85.7%)		8/9 (88.9%)		1.0
N0	6/13 (46.2%)	0.07	36/42 (85.7%)	0.58	**0.007**
N1	7/8 (87.5%)		14/18 (77.8%)		1.0
N2	8/9 (88.9%)		7/9 (77.8%)		1.0
M0	11/17 (64.7%)	0.14	44/55 (80.0%)	0.44	0.263
M1	6/6 (100%)		12/13 (92.3%)		1.0

Between cohorts, a significantly lower number of *BRAF*mut/MSS CIN positive cancers presented at early stages I/II compared to the *BRAF*wt/MSS cancers with CIN. This was evident overall (p = 0.02) and at individual regions 5q, 17p and 18q (p = 0.02, p = 0.04, p = 0.01 respectively). The rate of CIN positive cancers presenting at advanced stages (III/IV) was similar in both cohorts (Table 4).

Of the tumours with no lymph node involvement, 6/13 (46%) of *BRAF*mut/MSS cancers showed CIN compared to 36/42 (86%) of *BRAF*wt/MSS cancers (p = 0.007) (Table 4). *BRAF*mut/MSS cancers showed a trend for increasing rates of CIN coinciding with spread to at least one lymph node (p = 0.07). Although not significant, these CIN cancers also had a higher rate of metastases at 100%, compared to the number of CIN cancers without metastases at 65%. The *BRAF*wt/MSS cancers showed similar levels of CIN either with or without lymph node spread or metastases (Table 4).

Of the *BRAF*mut/MSS cancers showing co-occurrence of CIMP and CIN, a greater majority presented at late stages (III/IV) (10/16, 63%), compared to those presenting at early stages (I/II) (4/13, 31%), although this did not reach significance (Table 3 in Supplementary data S1).

Cox proportional hazard models were used to investigate overall cancer specific survival of patients with CIN cancers. To account for any confounding effects the variables of *BRAF* status (*BRAF*mut/MSS and *BRAF*wt/MSS) and stage (I/II and III/IV), along with all statistically significant higher order interactions, were included in the model. No significant relationships between presence of overall CIN and survival were demonstrated in either cohort (*BRAF*mut/MSS p = 0.81, *BRAF*wt/MSS p = 0.22). However, analysis at individual loci revealed the *BRAF*mut/MSS cancers with CIN at 18q and 8p to have significantly worse survival once adjusted for stage (p = 0.02, p<0.05 respectively) (Table 5). *BRAF*wt/MSS cancers showed no difference in survival rates regardless of CIN status at any individual loci (Table 5).

**Table 5:** Cox Proportional Hazard Models showing Hazard Ratio (HR) of Overall Survival for the interaction between *BRAF* and CIN for overall and individual genomic regions, with 95% Confidence Interval (CI) adjusted for stage (I/II and III/IV).

Genomic Region:	*BRAF*mut/MSS CIN+ Vs CIN−			*BRAF*wt/MSS CIN+ Vs CIN−		
	HR	95% CI	P Value	HR	95% CI	P Value
**Overall**	0.86	(0.25–3.00)	0.81	0.54	(0.20–1.44)	0.22
**5q**	2.95	(0.88–9.97)	0.08	0.76	(0.30–1.93)	0.56
**8p**	4.88	(1.03–23.08)	**0.046**	0.45	(0.15–1.39)	0.17
**17p**	1.21	(0.31–4.69)	0.78	0.62	(0.18–2.12)	0.44
**18q**	6.22	(1.32–29.19)	**0.02**	0.75	(0.29–1.96)	0.56

Patients with *BRAF*mut/MSS cancers had worse survival rates compared to those with *BRAF*wt/MSS cancers once adjusted for stage, regardless of presence or not of CIN (HR = 3.4, 95% CI [1.65, 6.99], p = 0.001).

## Discussion

Colorectal cancers arising via the serrated neoplastic pathway are characterised by a high frequency of *BRAF* mutation, MSI and CIMP. MSI cancers have been extensively studied, are diploid and confer an excellent prognosis. In the absence of MSI, a *BRAF* mutation correlates with a particularly poor patient outcome. This study reports that a high level of CIN is evident in *BRAF* mutant/MSS cancers and this demonstrates at least one mechanism of genomic instability by which these cancers form and progress. CIN was assessed with loss of heterozygosity (LOH) analysis with CIN assigned if at least one of the twelve markers was positive for LOH. The average fractional allelic loss (FAL) [38] which considers the number of markers showing LOH relative to the number of informative markers, was also calculated to further provide a quantifiable measure of the rate of CIN. Both methods found similarly high frequencies of CIN occurring in MSS cancers regardless of *BRAF* mutation status. Many of the CIN positive *BRAF* mutant/MSS cancers showed CIMP and thus this study challenges the belief that CIN and CIMP are mutually exclusive. It provides additional evidence that *BRAF* mutant/MSS cancers are fundamentally different to their MSI counterparts although they both potentially arise from *BRAF* mutant serrated polyps.

*BRAF* mutant/MSS cancers are an important subgroup of colorectal cancer due to their association with a particularly poor patient prognosis. However, due to their relatively low prevalence of approximately 5–7% of all colorectal cancers, the molecular mechanisms underlying this poor outcome have not been well studied. By identifying 60 *BRAF* mutant/MSS cancers from a large series of 1052 patients, the necessary power was attained to examine frequency of CIN in relation to other clinical and molecular variables. It is well established that *BRAF* mutant cancers predominantly occur in the proximal colon [12], [18], [37]. Certainly, the majority of *BRAF*mut/MSS cancers in this study were from this region (69%), however there was no difference in the rate of CIN found in proximal compared to distal cancers (Table 3; Table 2 in Supplementary data S1).

We have previously shown that *BRAF* mutant/MSS cancers have a comparable rate of *p53* mutation to *BRAF* wild type/MSS cancers, and this was significantly greater than the *p53* mutation rate found in *BRAF* mutant/MSI cancers. Furthermore, clinicopathological data between the *BRAF* mutant/MSS cancers and *BRAF* wild type/MSS cancers, also correlated in terms of a more advanced stage of presentation, comparable ages of onset, and equal gender distribution [37]. The extensive degree of CIN found in *BRAF* wild type/MSS cancers has been well documented. As *BRAF* mutant cancers are thought to derive from a serrated polyp, and *BRAF* mutant cancers that are microsatellite unstable are diploid; it may be postulated that *BRAF* mutant/MSS cancers would also be CIN-negative. However, based on our previous finding of multiple molecular and clinical similarities between the two MSS subgroups [37], we hypothesised that chromosomal instability would contribute to progression of a substantial proportion of *BRAF* mutant/MSS cancers. The data presented here support this hypothesis. We further hypothesise that in *BRAF* mutant cells, the acquisition of CIN is consistent with survival only in the absence of microsatellite instability. To find such similarly high rates of CIN occurring in a large cohort of *BRAF* mutant/MSS cancers provides further evidence of molecular similarities occurring between these two MSS subgroups although one may progress from serrated polyps, and the other from conventional adenomas.

The presence of CIN was examined over twelve markers spanning four chromosomal regions commonly deleted in colorectal cancer (chromosomes 5q, 8p, 17p and 18q). Although the overall rate of CIN was high in both *BRAF* mutant and wild type cohorts, examination of individual loci revealed differences between cohorts in the frequency of CIN events per loci. The *BRAF* mutant/MSS cancers had the greatest degree of CIN on the 8p chromosomal arm, and the least at the 18q region. In contrast, the *BRAF* wild type/MSS cancers had the highest frequency of instability on the 17p arm, and the least at 8p and 5q. A significant difference in the rate of CIN between the two groups was evident at the 18q chromosomal region with the *BRAF* wild type/MSS cancers having significantly greater loss at the 18q chromosomal region. Chromosome 18q21.1 harbours the tumour suppressor genes *SMAD4, SMAD2* and *deleted in colorectal* cancer (*DCC*) genes. The *SMAD4*/*2* gene complex is involved in signal transduction of the TGF-beta pathway and particularly in early stages of tumourigenesis can regulate the expression of target genes resulting in arrested growth and apoptosis [39], [40]. Within the MAPK kinase pathway, aberrant activation of downstream effectors of *BRAF* have been found to modulate TGF-beta mediated signalling of *SMAD*s [39], [41], where *ERK* has been implicated in inhibiting *SMAD* nuclear translocation [42]. As *BRAF* mutant/MSS cancers have a constitutively active MAPK pathway, loss of the *SMAD* loci may be redundant and could help to explain the significantly lower rate of deletion found at the 18q region in these compared to *BRAF* wild type/MSS cancers. *DCC* is subject to epigenetic silencing [43] and may be targeted by this mechanism rather than deletion in *BRAF* mutant cancers. Overall, differences in the rate of CIN found at individual regions, indicate that certain loci are preferentially deleted to inactivate the residing tumour suppressor gene according to the genetic background of the cancer.

A loss event of one allele is typically preceded or followed by a mutational event of the second allele at the same region to inactivate the target tumour suppressor gene. This was evident in the present study where in both *BRAF* mutant and wild type cancers, *p53* mutation correlated with CIN at 17p. Average FAL scores were recalculated based on chromosomes 5q, 8p and 18q in order to remove the influence of 17p CIN on *p53* mutation rate. Mutation of *p53* significantly correlated with a higher FAL score for CIN in *BRAF* wild type cancers. A similar but non-significant trend was observed in *BRAF* mutant cancers. These data support our hypothesis that *BRAF* mutant cancers are not entirely distinct from those arising via the traditional adenoma-carcinoma pathway, but may share multiple molecular features required for tumour progression.

It has been postulated that CIN and CIMP are mutually exclusive [29], [30]. Interestingly, this study clearly demonstrates a high frequency of CIN and CIMP co-occurrence in the *BRAF* mutant/MSS cancers (72%), which suggests these forms of genetic and epigenetic instabilities can coexist within this molecular background. These findings highlight the necessity of stratification to identify molecular features of important cancer subgroups. An inverse relationship between CIN and CIMP has previously been observed in a predominantly *BRAF* wild type cancer cohort where only 9 *BRAF* mutant/MSS cancers were investigated [29]. A low level of CIN has been found in CIMP positive cancers using a genome wide array approach, however *BRAF* mutational status was not assessed [44]. The lack of association reported in these studies likely reflects the low frequency of CIMP in *BRAF* wild type cancers. In the present study, CIMP occurred in only 4% of *BRAF* wild type cancers, precluding the meaningful analysis of CIMP correlating with CIN in this subgroup.

In *BRAF* mutant/MSS cancers, significantly increasing levels of overall CIN were found with advanced stages of presentation (Tables 3 and 4), suggesting that CIN may contribute to progression of this type of cancer. This pattern was also observed at the chromosomal regions of 18q and 17p (Table 4). A considerable rate of CIN at the 8p region occurring at both early and late stages, may account for this region showing the greatest rate of CIN within this cancer type, and furthermore, may implicate a role for loss of an 8p tumour suppressor gene early in disease development.

In contrast, for overall CIN and at all individual chromosomal regions, the *BRAF* wild type/MSS cancers showed comparable rates of CIN at early and late stages, and this suggests that CIN is important for initiation or early progression within this cancer subgroup. The higher average fractional allelic loss (FAL) score observed for *BRAF* wild type versus mutant MSS cancers may reflect the earlier onset of CIN and therefore greater accumulation of CIN events (Table 3). In *BRAF* mutant/MSS cancers, the rate of CIN increased with increasing lymph node involvement and metastases although this did not reach statistical significance (Table 4). This pattern was not observed in the *BRAF* wild type cancers. A potentially synergistic relationship between BRAF mutation, CIN and tumour progression requires further investigation.

The mechanism underlying the association of CIN with advanced stage in *BRAF* mutant/MSS cancers requires elucidation. It is possible that early activation of the MAPK pathway is sufficient to initiate tumourigenesis. Tumour progression may then be promoted by factors underlying CIN such as disruption of mitotic spindle checkpoints and/or centrosome regulators, which may contribute to the particularly aggressive phenotype of this cancer subgroup. For example, overexpression of the Aurora kinases (*AURKA* and *AURKB*) have been associated with centrosome amplification and chromosomal mis-segregation [45]. In CRC, overexpression of *AURKA* is associated with CIN [46], while *AURKB* correlates with advanced stages [47] and a worse patient outcome in many cancer types [48], [49]. Increased levels of *AURKB* have also been associated with activation of the MAPK pathway in melanoma, and furthermore application of the *BRAF* inhibitor vemurafenib, abrogated *AURKB* expression [50]. This suggests that Aurora kinase family members, particularly *AURKB*, may play a role in mediating CIN in advanced *BRAF* mutant/MSS cancers. Additionally, in *BRAF* mutant/MSS cancers there was a greater rate of co-occurrence of CIMP and CIN in late compared to early stages (63% and 31% respectively) (Table 3 in Supplementary data S1). This may suggest that the compounding effects of both chromosomal and epigenetic instabilities could contribute to the aggressive phenotype and unfavourable outcomes observed in patients with a *BRAF* mutant/MSS cancer. As expected there were too few CIMP high cancers in the *BRAF* wild type/MSS cohort to allow for comparisons between cohorts.

Overall patient survival assessed with Cox proportional hazard models, adjusted for stage, indicated that *BRAF* mutant/MSS cancers with CIN at the 18q and 5q loci, conferred significantly poorer outcomes compared to those *BRAF* mutant/MSS cancers without CIN at these loci (Table 5). This may imply that CIN at certain genomic regions contributes to the worse survival of *BRAF* mutant/MSS cancers. CIN at 18q and 8p has previously been associated with a poor prognosis and worse overall survival [31], [51], [52], [53], [54]. However, loss at 18q relating to poor survival has been disputed in other studies [32], [55]. The data presented here supports the hypothesis that a poorer prognosis may be driven by CIN at defined chromosomal locations in MSS cancers bearing a *BRAF* mutation.

This discussion has eluded to possible molecular interactions that could facilitate the acquisition of CIN in these cancers. In accordance with previous findings [20], [22], the *BRAF* mutant/MSS cancers conferred poorer survival rates compared to the *BRAF* wild type cancers, irrespective of the presence or not of CIN (p = 0.001). The molecular mechanisms of how a *BRAF* mutation on a background of MSS confers such a detrimental patient outcome is currently unclear. *BRAF* mutant/MSS cancers are an aggressive cancer type and this may be related to the findings that a *BRAF* mutation correlates with overexpression of the angiogenic factor, *VEGFA*, in colorectal cancer [56]. Inducible *Raf* has been found to cause an invasive phenotype dependent on activation of the TGF-beta pathway [57] which develops a more proliferative role in advanced tumourigenesis [39], [40]. Furthermore, activated *Raf* can contribute to the epithelial-mesenchymal transition [57] which hallmarks the initiation of metastasis and may help to explain the aggressive nature of the *BRAF* mutant/MSS cancers. In contrast, the *BRAF* mutation on a background of MSI relates to a comparably favourable patient outcome. This may be due in part to the lower rate of *VEGFA* found in MSI compared to MSS CRCs [58] which may reduce the level of metastatic spread in MSI cancers. MSI cancers commonly have mutant *TGFBR2* [59] which may negate the proliferative effects of the TGF-beta pathway and impair the epithelial to mesenchymal transition in MSI compared to MSS cancers [60].

Recent studies utilizing array based methodologies have found novel regions of loss and/or gain events associating with worse prognoses and disease progression [26], [61]. Furthermore, recent array based investigations have found MSI cancers with DNA copy number changes in unique regions of the genome [61], [62], [63], as well as copy neutral LOH events [64], [65]. By furthering this type of experimental approach, the relationship between MSI and CIN will be better understood.

This study, for the first time, reports a high frequency of CIN in the aggressive *BRAF* mutant/MSS cancers thought to progress via the serrated neoplastic pathway. Although similarly high rates of CIN were observed in *BRAF* mutant and wild type MSS cancers, differences existed between the two cohorts regarding the chromosomal regions most commonly targeted, and the stages of presentation at which CIN was most frequent. Additionally, we report on the novel finding of both CIN and CIMP co-occurring in *BRAF* mutant/MSS cancers, and potentially the combined impact of such may contribute to the detrimental outcome for patients with this cancer type.

Findings from this and our previous study [37], suggest that *BRAF* mutant/MSS cancers form an aggressive cancer subgroup with distinct clinical and molecular features. Extended investigations into the histology and molecular characteristics of these *BRAF* mutant/MSS cancers will help to reveal further potential causes of the worse prognosis observed in this cancer subgroup. Importantly, this could ultimately aid the identification of therapeutic targets to treat this aggressive form of colorectal cancer.

## Supporting Information

Supplementary data S1 (DOCX)
