# Supplementary material for: Chromosomal Instability in BRAF Mutant, Microsatellite Stable Colorectal Cancers
Source: PLoS One. 2012 Oct 22;7(10):e47483. doi: 10.1371/journal.pone.0047483 (PMC3478278; doi:10.1371/journal.pone.0047483)
Supplement: Supplementary data S1 — (DOCX) [file pone.0047483.s001.docx]

**Supplementary Data:** (Chromosomal Instability in BRAF Mutant, Microsatellite Stable Colorectal Cancers)

**Loss of Heterozygosity Analysis - Details of PCR:**

Cancer and corresponding normal DNA were amplified with PCR using either x1 unit DyNazyme taq (Finnzymes; Espoo, Finland) or x1 unit Gold Taq (Roche; Basel, Switzerland), with corresponding x1 PCR Buffer and 1.5-2.5mM MgCl_2_; 1mM dNTPs (Promega; Madison, Winsconsin); 20uM forward and reverse primer (Sigma Aldrich; St Louis, Missouri); and 37MBq dATP α-^33P^ (Perkin Elmer; Boston, Massachusetts). PCR cycles varied slightly depending on the marker analysed as outlined in table below.

**Supplementary Data. Table 1:** Summary of microsatellite marker PCR conditions for LOH analysis:

| LOH Marker | Taq | MgCl_2_ (mM) | PCR Annealing Temperature (T_A_) (°C) and Number of Cycles |
| --- | --- | --- | --- |
| D5S346 | Gold | 1.5 | T_A_ 62-56°C x35; 55° x3 |
| D5S1466 | Gold | 1.5 | T_A_ 62-56°C x35; 55° x1 |
| D5S489 | Gold | 1.5 | T_A_ 62-56°C x35; 55°C x1 |
| D8S254 | Gold | 1.5 | T_A_ 62-56°C x35; 55°C x4 |
| D8S258 | Gold | 1.5 | T_A_ 64-58°C x 35; 57° x3 |
| D8S1121 | Gold | 1.5 | T_A_ 60°C x33 |
| D17S926 | Gold | 1.8 | T_A_ 65-58$^{\circ}$C x35; 57°C x3 |
| D17S261 | DyNazyme | 2.5 | T_A_ 55°C x40 |
| D17S578 | Gold | 1.5 | T_A_ 64-56°C x30; 55° x10 |
| D18S460 | DyNazyme | 2.5 | T_A_ 60-56°C x10; 55°C x35 |
| D18S487 | DyNazyme | 1.8 | T_A_ 60-55°C x12; 55°C x40 |
| D18S55 | DyNazyme | 2.5 | T_A_ 55°C x40 |

**Supplementary Data. Table 2:** Clinicopathological Characteristics Relative to presence or not of Chromosomal Instability (CIN)

| CIN + or CIN – and Clinicopathological Characteristic | BRAFmut/MSS (Serrated) | P value | BRAFwt/MSS (Traditional) | P Value | |
| --- | --- | --- | --- | --- | --- |
| Location: |  |  |  |  | |
| CIN +\| Proximal | 23/31 (74.2%) | 1.0 | 18/25 (72.0%) | 0.11 | |
| CIN - \| Proximal | 8/31(25.8%) |  | 7/25 (28.0%) |  | |
| CIN +\| Distal | 12/15 (80.0%) |  | 50/57 (87.7%) |  | |
| CIN - \| Distal | 3/15 (20.0%) |  | 7/57 (12.3%) |  | |
| Av Age of Onset (yrs): |  |  |  | |  |
| CIN + | 67.6 | 0.62 | 67.9 | | 0.61 |
| CIN – | 65.6 |  | 69.9 | |  |
| Gender : |  |  |  |  | |
| CIN +\|Female | 20/29 (69.0%) | 0.77 | 33/39 (84.6%) | 0.78 | |
| CIN - \| Female | 9/29 (31.0%) |  | 6/39 (15.4%) |  | |
| CIN +\| Male | 21/28 (75.0%) |  | 41/51 (80.4%) |  | |
| CIN - \| Male | 8/28 (25.0%) |  | 10/51 (19.6%) |  | |

**Supplementary Data. Table 3:** Presence of CIN and CIMP relative to AJCC Stage at Presentation

|  | BRAF mut/MSS (Serrated) | | | BRAF wt/MSS (Traditional) | | |
| --- | --- | --- | --- | --- | --- | --- |
|  | **Early Stage (I/II) n=13** | **Late Stage (III/IV) n=16** | P value | **Early Stage (I/II) n=35** | **Late Stage (III/IV) n=32** | P value |
| CIN+ \|CIMP high | 4/13 (30.8%) | **10/16 (62.5%)** | 0.14 | 1/ 35 (2.9%) | 0 | 1.0 |
| CIN+ \| CIMP 0/low | 2/13 (15.4%) | 4/16 (25.0%) | 0.66 | 29/35 (82.9%) | 27/32 (84.4%) | 1.0 |
| CIN- \|CIMP high | 3/13 (23.1%) | 0 |  | 0 | 0 |  |
| CIN- \| CIMP 0/low | 4/13 (30.8%) | 2/16 (12.5%) | 0.36 | 5/35 (14.3%) | 5/32 (15.6%) | 1.0 |
